# Domestic violence against women in Eastern Sudan

**DOI:** 10.1186/1471-2458-14-1136

**Published:** 2014-11-04

**Authors:** AbdelAziem A Ali, Khalid Yassin, Rawia Omer

**Affiliations:** Faculty of Medicine, Kassala University, Kassala, Sudan; Faculty of Medicine, Alnelain University, Khartoum, Sudan; Ministry of Health, Kassala State Kassala, Sudan

**Keywords:** Violence, Women, Public, Health, Sudan

## Abstract

**Background:**

Violence against women is one of the major public health problems in both developed and developing worlds. The aim of this study was to investigate the prevalence of current (occurred in one year preceding the survey) domestic violence and socio-demographic factors associated with domestic violence against women.

**Methods:**

This was a cross sectional household survey (face to face interview) conducted in Kassala, eastern Sudan, from 1^st^ March to 1^st^ June 2014. Multivariable analyses were performed, Confidence intervals of 95% were calculated and *P <* 0.05 was considered significant.

**Results:**

Of the 1009 women, 33.5% (338) reported current experience of physical violence and, of these 338 women, 179 (53%) and 159 (47%) reported moderate and severe form of physical violence respectively. The prevalence of sexual coercion, psychological violence and verbal insult was 17% (172\1009), 30.1% (304\1009) and 47.6% (480\1009) respectively. In the majority of cases, violence was experienced as repeated acts, ie, more than three times per year. For verbal insult 20.1% (203\480) and 27.5% (277\480) reported yelling and shouting respectively. Again 251 (24.9%) and 270 (26.8%) women reported that they experience divorce threat and second marriage threat respectively. In logistic regression model, husband’s education (OR = 1.5; CI = 1.0-2.1; *P* = 0.015), polygamous marriage (OR = 1.9; CI = 1.3-2.9; *P* = 0.000), and husband’s alcohol consumption (OR = 13.9; CI = 7.9-25.4; *P* <0.000) were significantly associated with domestic violence.

**Conclusions:**

Domestic violence was found to be highly prevalent in eastern Sudan and strongly associated with the educational status, polygamous marriage and husband’s alcohol consumption. We recommend more research to include men.

## Background

The World Health Organization (WHO) defined domestic violence as “the range of physically, psychologically and sexually coercive acts used against adult and adolescent women by current or former male intimate partners” [1]. Violence against women is one of the major public health problems in both developed and developing worlds. It varies from community to community and the pattern of the violence affected by the different socioeconomic and cultural factors [2]. Worldwide the prevalence of domestic violence against women ranges between 15% and 71% [3]. Data on domestic violence is scarce particularly in developing countries. Many theories can be used to explain the occurrence of domestic violence against women; these include sociological, gender and family system theories [4, 5]. Sociological theories indicate that low level of education, economic vulnerability, stress and a closed social network increase the risk of partner violence, while the gender theory is indicated by the male being the dominant person in some communities. On the other hand family system theories focus on communication, relationship and problem solving skills of couples in whom violence occurs [4, 5].

There is limited data on the prevalence of the domestic violence against women in the sub-Saharan and African countries [6]; however there are a recognized correlation between the domestic violence and various reproductive health problems such as non use of contraception and sexually transmitted diseases [7]. Domestic violence is against the religious perception and the Islamic teaching discourages the violence against women. Sudan is one of the largest African countries with different public health problems such as female genital mutilation and teenage pregnancy [8, 9]. A number of steps have been taken by the government of Sudan to fight violence against women and girls, and it is extremely important to consider them. In 2005 a state plan to combat violence against women was adopted and a new Violence against Women (VAW) Unit was created at the Ministry of Justice [10]. To our knowledge no available data on domestic violence against women in Sudan exists, thus the current study designed and directed to investigate the prevalence rate and socio-demographic factors associated with domestic violence against women in Eastern Sudan aiming to provide the policy makers with fundamental data to reduce the prevalence rate of this practice.

## Methods

### Study area and period

This was a community-based cross sectional household survey conducted in Kassala, eastern Sudan from 1^st^ March to 1^st^ June 2014. Kassala is located in eastern Sudan, 600 km from Khartoum; it is 42,282 square km, with a population of 1.8 million people. Of these, 440,491 women are of reproductive age. Kassala state bordered two neighboring countries, Eritrea and Ethiopia, with a prominent diversity in culture, religion, language and ethnicity. The state comprises of 6 administrative localities, the responsibilities of the maternal health care services lie with the department of the reproductive health at the ministry of health. There are one referral (tertiary) hospital, two secondary care level hospitals, 13 rural hospitals and 144 primary health centers providing preventive and curative maternal health care services. Kassala state is described by high prevalent rate of illiteracy, child marriage and female genital mutilation [8].

### Eligibility criteria

The study population was all women in Kassala during the study period. Women were eligible to participate in the study if they resided in the study period and they were in the reproductive age (15 – 49 years), also women were eligible if they married for ≥ one year and agreed to participate in the study. A total sample size of 1009 women was calculated to be needed using a single population proportion formula, which would provide 80% power and which assumed 10% of women would not respond. Multi-stage sampling was used to select the study population. Households were selected by systemic random sampling of the sampling frame obtained from the administrate unit in Kassala. When more than one eligible woman was found in a selected household, one of the women was interviewed randomly. If no eligible woman was encountered in the selected household, the next household on the right was visited.

### Data collection and variables

Ten medical students were selected as interviewers; they had been trained in relationship building, local cultures, language, confidentiality and how to administer the questionnaires. For more security we interviewed the participant during the day activity in the absence of their husbands and this point was considered in the ethical clearance. After obtaining written consent, self structured questionnaires were used to gather data (face to face interview) from the eligible women. We used our own questionnaire which was constructed by the authors to consider different individual issues specific to our environment such as polygamous marriage, threat with divorce and second marriage however the violence items stem from validated and widely used questionnaire (Domestic Violence Questionnaire in Married Women) [11]. We started to fill the questionnaire after explanation of the purpose of the study and making sure the aim of the study was well understood by the participant. Information sought in the questionnaire included socio-demographic characteristics (age, ethnicity, educational level, residence, duration of marriage, parity, occupation, consanguinity, polygamous marriage, number of family members, husband’s age, husband’s education, occupation and husband’s alcohol consumption). We aimed to investigate the prevalence of current (occurred in one year preceding the survey) domestic violence and socio-demographic factors associated with domestic violence against women in eastern Sudan. This violence included physical violence, sexual coercion, psychological violence and verbal insult. Physical violence was measured as moderate (slapping, throwing things, pushing, shoving) or severe (hitting, kicking, dragging, beating, chocking, burning) and sexual coercion was defined as being coerced to perform sexual acts against woman’s will and physically forced into sexual intercourse by the husband. Psychological violence was defined as insulting the woman or making her feel bad about herself, belittlement or humiliation in front of others, doing things to scare or intimidate her on purpose, and threats to hurt her or someone she cared about and the verbal insult included yelling, shouting, threat with divorce or\and second marriage.

### Statistical analysis

Data were entered into a computer database and SPSS software (SPSS Inc., Chicago, IL, USA, version 16.0). Multivariable analyses were performed. Domestic violence against women was the dependent variable and socio-demographic characteristics were independent variables. Confidence intervals of 95% were calculated and *P <* 0.05 was considered significant.

### Ethics

The study approved and received ethical clearance from the Research Board at the Ministry of Health, Kassala State, Eastern Sudan. In this study we followed the WHO guidelines for ethical clearance and written consent. The written consent included the research title, purpose, benefit, participants’ right, confidentiality and signature.

## Results

### Participants’ characteristics

A total of 1009 women were enrolled and none of them refused to participate in this study. Their mean (SD) age, husband’s age and parity was 32.9 (8.1), 42.4 (10.4) and 3.2 (2.4) respectively. The duration of their marriage ranged between 1 to 27 years. Three-quarters of the women (757\1009, 75%) lived in urban community and more than one-third of the women (197\1009, 33.5%) lived in a polygamous marriage. Almost half (500\1009, 49.6%) of the women were of less than secondary level education and 66.1% (667\1009) were housewives.

### Prevalence of domestic violence

Of the 1009 women, 33.5% (338) reported current (in one year preceded the survey) experience of physical violence and, of these 338 women, 179 (53%) and 159 (47%) reported moderate and severe form of physical violence respectively, Table 1. Among physical, sexual and psychological violence the most commonly occurring single form was physical violence (33.5%). The prevalence of sexual coercion, psychological violence and verbal insult was 17% (172\1009), 30.1% (304\1009) and 47.6% (480\1009) respectively. In the majority of cases, violence was experienced as repeated acts, ie, more than three times per year, Table 2. For verbal insult 20.1% (203\480) and 27.5% (277\480) reported yelling and shouting respectively. Again 251 (24.9%) and 270 (26.8%) women reported that they experience divorce threat and second marriage threat respectively.

**Table 1:** **Different forms of physical violence among women in Eastern Sudan, 2014**

Form of violence	Number	Percentage of the total ( ***n = 338*** )
***Moderate forms***		
**Slapping**	**86**	**25.4%**
**Throwing things**	**60**	**17.8**
**Pushing**	**29**	**8.6**
**Shoving**	**4**	**1.2**
***Severe forms:***		
**Hitting**	**43**	**12.7**
**Kicking**	**14**	**4.1**
**Dragging**	**32**	**9.5**
**Beating**	**40**	**11.8**
**Chocking**	**20**	**5.9**
**Burning**	**10**	**3**
**Total**	**338**	**100**

Data was shown as number and percentage as applicable.

**Table 2:** **Frequency of physical violence per year among women in Eastern Sudan 2014**

Frequency	Number	Percentage of the total ( ***n = 338*** )
**Once**	**71**	**21**
**Twice**	**52**	**15.4**
**Thrice**	**44**	**13**
**>Thrice**	**171**	**50.6**
**Total**	**338**	**100**

Data was shown as number and percentage as applicable.

### Factors associated with domestic violence

Among the socio-demographic data there were commonalities and difference in the factors associated with the different type of domestic violence against the surveyed women. In logistic regression model, husband’s education (OR = 1.5; CI = 1.0-2.1; *P* = 0.015), polygamous marriage (OR = 1.9; CI = 1.3-2.9; *P* = <0.001), and husband’s alcohol consumption (OR = 13.9; CI = 7.9-25.4; *P* <0.00) were significantly associated with physical violence, Table 3. While psychological violence was significantly varied with women’s education (OR = 1.2; CI = 0.9-1.2; *P* = 0.010), polygamous marriage (OR = 1.6; CI = 1.1-2.4; *P* = 0.006) and husband’s alcohol consumption (OR = 4.5; CI = 2.8-7.2; *P* = <0.001) there was significant statistical association between sexual coercion husband’s age (OR = 1.0; CI = 1.0-1.1; *P* = 0.031), polygamous marriage (OR = 1.7; CI = 1.1-2.7; *P* = 0.009) and husband’s alcohol consumption (OR = 5.6; CI = 3.5-9.1; *P* = <0.001), Table 4.

**Table 3:** **Factors associated with physical violence against women in Eastern Sudan using multivariable analyses, 2014**

Variable	Multivariable analyses		
	OR	95% CI	***P*** -value
Women’s age, years	0.1	0.9-1.0	0.604
Parity ≥3	0.1	0.9-1.1	0.302
Husband’s age, years	0.1	0.9-1.0	0.165
Duration of marriage ≥5 years	0.9	0.9-1.0	0.646
1^st^ degree relationship	1.2	0.9-1.7	0.120
Women's education < secondary level	1.2	0.8-1.7	0.248
Women’s occupation, housewives	0.8	0.6-1.0	0.231
Husband’s education < secondary level	1.5	1.0-2.1	0.015
Rural residence	1.0	0.7-1.4	0.879
Ethnicity, hadandwa tribe	1.0	0.8-1.2	0.635
Family members ≥5	0.9	0.8-1.0	0.263
Polygamous marriage	1.9	1.3-2.9	<0.001
Husband’s alcohol consumption	13.9	7.6-25.4	<0.001

*Abbreviations: OR* Odds Ratio, *CI* confidence interval.

**Table 4:** **Factors associated with psychological insult and sexual coercion against women in Eastern Sudan using multivariable analyses, 2014**

	Psychological insult			Sexual coercion		
Variable	OR	95% CI	***P*** -value	OR	95% CI	***P*** -value
Women’s age, years	1.0	0.9-1.0	0.539	1.0	0.9-1.0	0.256
Parity ≥3	1.0	0.9-1.0	0.848	1.0	0.9-1.1	0.419
Husband’s age, years	1.0	0.9-1.0	0.121	1.0	1.0-1.1	0.031
Duration of marriage ≥5 years	0.9	0.9-1.0	0.768	0.9	0.9-1.0	0.102
1^st^ degree relationship	1.2	0.9-1.7	0.097	1.2	0.8-1.8	0.216
Women’s education < secondary level	1.5	1.1-2.2	0.010	1.1	0.7-1.7	0.527
Women’s occupation, housewives	0.8	0.7-1.0	0.230	1.0	0.8-1.3	0.738
Husband’s education < secondary level	1.3	0.9-1.8	0.088	1.4	0.9-2.1	0.084
Rural residence	1.1	0.8-1.6	0.435	1.0	0.6-1.6	0.798
Ethnicity, hadandwa tribe	1.0	0.8-1.3	0.495	1.0	0.8-1.3	0.755
Family members ≥5	0.8	0.8-1.0	0.441	0.8	0.9-1.0	0.166
Polygamous marriage	1.6	1.1-2.4	0.006	1.7	1.1-2.7	0.009
Husband’s alcohol consumption	4.5	2.8-7.2	<0.001	5.6	3.5-9.1	<0.001

*Abbreviations: OR* Odds Ratio, *CI* confidence interval.

## Discussion

To our knowledge this is the first study to investigate domestic violence against women in Sudan. Domestic violence against women is extremely sensitive issue in our community and it was anticipated to find underestimated prevalence rate. The data was collected by female students who lived in the same state and were well respected and trusted in the community. The current physical violence which this study describes (33.5%) is comparable with other results from some Asian countries such as Vietnam and India [12]. A report from Iran of 2400 married women found that 15% experienced physical violence from their husbands in the previous year, 42% sexual coercion, and 82% various degrees of psychological abuse [13]. According to the World Health Organization’s multi-country study on violence against women in intimate relationships, the lifetime prevalence of physical or sexual coercion ranges between 15% and 71%, and past-year prevalence also shows a wide variation (4%–54%), with the lowest rates found for Japan and the highest for Ethiopia, Peru, and Bangladesh [3].

Our findings indicate poor socio-economic circumstances contribute significantly to domestic violence against women. Sudan is a low-income country with more than 30 million inhabitants. It is a male-dominated society, where husband violence is accepted as a cultural norm and viewed as normal behavior within the families. Although it’s against Islamic teachings, domestic violence against wives is often justified by the woman’s misbehaviour or disobedience. Moreover in developing countries other factors may be attributed to high prevalence of violence, these include poverty and fear of the mother from divorce because divorce is a social stigma for the mother in our community. Another factor is the social norm which strongly encourages the women to accept the different misbehaviors of the husband. Our findings showed unacceptably high prevalence of psychological insult and sexual coercion and it is of no doubt the different forms of violence give negative impact on the women’s life in particular their care after children and community participation [14]. Also our respondents experienced different forms of insults, second marriage threat, verbal and divorce threats. Females are usually the victims in male-dominated societies with less gender equality like Sudan. It is not of great surprise to find high prevalence rate of sexual coercion, psychological insults or other form of violence in the community where there is high prevalence of physical violence. Research has shown that physical violence is often associated with psychological or and sexual coercion [15]. Our results showed significant association between domestic violence against women, husband’s alcohol consumption and polygamous marriage. Women living in polygamous marriage and with alcoholic partners expressed greater incidence of troubles and conflict with their husbands and this put them at greater risk of violence. This fact possibly might interpret the association between domestic violence and these two variables. In consistent with study conducted in Pakistan [12], the multivariable analysis in our study showed strong association between husband education and physical violence and between women’s education and psychological violence against women, this is in line with what was also been found in other public health study in Bangladesh [16].

We believe that the findings of the current study will help the policy makers to put their strategic plan to fight the practice. Domestic violence was found to be associated with husbands’ education, polygamous marriage and husband’s alcohol consumption however, causality cannot be determined due to the cross sectional design of the study. The prevalence of domestic violence is possible to be reduced by improving the educational status of the community, advice against alcohol consumption and create a governmental law for the punishment concerning the violence against women. Although the participation was optional, no woman refused to participate in this study, this of no doubt strengthen our findings.

### Limitations

One of the major limitations of this study we asked the women about the current domestic violence (one year preceded the survey) rather than the past life violence. The past-year prevalence rates provide more robust estimates due to the fact that these events have taken place closer in time compared with events that have taken place further in the past and which are easier to forget. Our study is a cross sectional one thus the causality between domestic violence and the associated factors cannot be determined. Furthermore violence in developing and male-dominated societies might occur against daughters and other close relatives; however our study investigated the husband violence against their wives. Also the men were not investigated to understand the magnitude of the problem and why they practice domestic violence against their wives.

## Conclusions

Domestic violence was found to be highly prevalent in eastern Sudan and strongly associated with the educational status, polygamous marriage and husband’s alcohol consumption. We recommend more research to include men.
